# BioMove: Biometric User Identification from Human Kinesiological Movements for Virtual Reality Systems

**DOI:** 10.3390/s20102944

**Published:** 2020-05-22

**Authors:** Ilesanmi Olade, Charles Fleming, Hai-Ning Liang

**Affiliations:** 1 Department of Computer Science and Software Engineering, Xi’an Jiaotong-Liverpool University, Suzhou 215123, China; Ilesanmi.Olade@xjtlu.edu.cn; 2 Department of Computer Science, University of Liverpool, Liverpool L69 7ZXl, UK; 3 Department of Computer and Information Science, University of Mississippi, Oxford, MS 38677, USA; fleming@olemiss.edu

**Keywords:** biometrics, user Identification, usability, security, virtual reality, kinesiology, mobility

## Abstract

Virtual reality (VR) has advanced rapidly and is used for many entertainment and business purposes. The need for secure, transparent and non-intrusive identification mechanisms is important to facilitate users’ safe participation and secure experience. People are kinesiologically unique, having individual behavioral and movement characteristics, which can be leveraged and used in security sensitive VR applications to compensate for users’ inability to detect potential observational attackers in the physical world. Additionally, such method of identification using a user’s kinesiological data is valuable in common scenarios where multiple users simultaneously participate in a VR environment. In this paper, we present a user study (n = 15) where our participants performed a series of controlled tasks that require physical movements (such as grabbing, rotating and dropping) that could be decomposed into unique kinesiological patterns while we monitored and captured their hand, head and eye gaze data within the VR environment. We present an analysis of the data and show that these data can be used as a biometric discriminant of high confidence using machine learning classification methods such as kNN or SVM, thereby adding a layer of security in terms of identification or dynamically adapting the VR environment to the users’ preferences. We also performed a whitebox penetration testing with 12 attackers, some of whom were physically similar to the participants. We could obtain an average identification confidence value of 0.98 from the actual participants’ test data after the initial study and also a trained model classification accuracy of 98.6%. Penetration testing indicated all attackers resulted in confidence values of less than 50% (<50%), although physically similar attackers had higher confidence values. These findings can help the design and development of secure VR systems.

## 1. Introduction

The use of virtual reality (VR) in a variety of applications has increased significantly in the last few years. The availability of inexpensive VR hardware together with rapid advances in their display and interaction peripherals have made VR popular. VR is currently being used in numerous domains such as medical-care [1], education [2], advertisement [3], shopping [4,5] and manufacturing [6]. High-end VR products are striving towards being fully wireless, reduced size and improved personalization. These new features will open up a lot of usable possibilities and application domains. This new hardware also offers new possibilities to authenticate or identify users, other than traditional ways to login, such as via PIN or other popular tethered methods [7]. In this research, we provide the first approach to identifying users from a natural kinesiological aspect in VR environments while including head-mounted-display (HMD) integrated eye-tracking and gesture controllers. This achieved an accuracy of 98.6%, which is significantly higher than other approaches that authenticate users in real-world environments from sparse trajectories obtained using non-invasive or body-mounted sensors (e.g., [8]), which is discussed in the Related Works section. There will be a strong demand for a robust, transparent, easy-to-use and non-intrusive identification approach that allows these VR devices to be securely used by multiple users without any perceived or actual inconvenience. As VR applications continue to grow, one aspect that has received little attention, to the best of our knowledge, is privacy and security issues of VR systems. User interaction in a VR environment is very different from other interactive systems, such as mobile phones or computers [9,10,11]. Various well understood security threats and attacks work in a completely different fashion within a VR environment, which makes well-known defense mechanisms ineffective or inapplicable. For example, directly implementing PIN or SWIPE authentication in VR environments, without any modifications, exposes the users to visual or observational attacks to a much larger extent than non-VR environments because of the relatively large movements of interactions in VR, and because most VR devices obscure the user’s view, limiting their awareness of the external environment. These types of identification mechanisms will not only be impractical for VR systems but also does not leverage their unique and inherent features and hardware capabilities.

Our research focuses on the level of security needed to positively confirm the identity of a user using various known attributes that are distinct from other users engaged in a VR environment. In VR systems, users’ body motion is captured to provide an immersive experience where their actions can be mimicked by avatar representations in real-time [12]. This mimicry can range from the movements of their head, hand, leg and other parts, including eyes when an eye tracker is present in the VR device [10,11,12,13]. VR systems endeavor to present these movements that are as kinesiologically closed as possible to the users’ movements [14,15,16]. Kinesiology is the study of the mechanics of body movements. A large body of literature exists on kinesiology [17,18,19], and its results have been used by various disciplines including health, criminology, sports, rehabilitation and identification of people. Kinesiology defines and identifies co-joined movement behavioral patterns by the user’s head and other limbs as an indication of specific movements in progress or about to start. For example, a user reaching to the ground to pick up an item must have the knees or back bent while the head may face the particular direction according to the intended action. We argue that the kinesiological behavioral patterns exhibited by a user while interacting with a VR system to perform activities contain unique identifying cues that provide some measure of biometric confidence about the person using the VR environment. If these cues are reliably captured, using inertial and orientational sensors that now come in the VR devices, the user’s movements can be recorded and used to create a biometric profile, which could then be used to provide an extra layer of security for the sensitive processes carried out in the VR space. It is possible to envision a transparent, easy-to-use and non-intrusive identification method that may be used independently or as a second factor with a pre-existing VR identification system. We believe other studies (e.g., [20,21]) exploring the migration of standard mobile device identification systems such as PIN and PATTERN into virtual reality environments will benefit from an additional non-intrusive identification system.

In this paper, we present BioMove, a behavioral pattern identification system that explores our above stated hypothesis. In particular, we investigate whether the head, limb, torso and eye movement patterns displayed by the user in the virtual reality space could be used as a biometric authenticating factor. To test the above hypothesis, we performed the following three activities.

**Figure 1:**
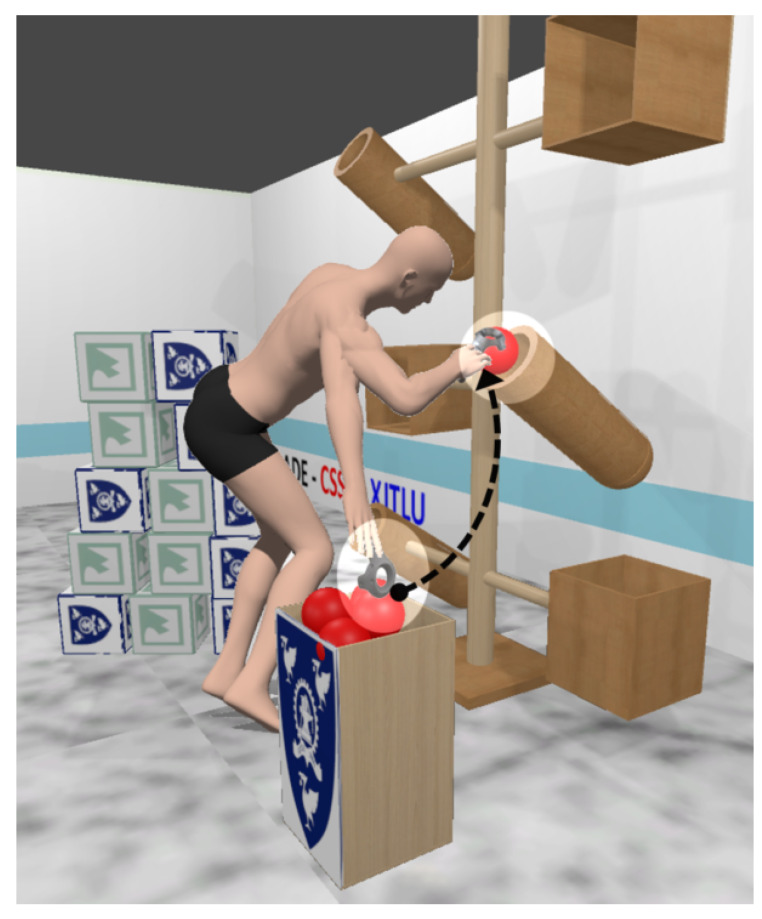
A screenshot of the task based VR environment. The environment was designed to elicit task based movements of users that allowed for biometric identification. Participants would perform movements that were primitively elliptical. In the above example, the user needed to relocate the ball from the bin to the container.

1. Kinesiologically designed VR environment: We implemented a carefully designed VR environment to ensure the observation and recording of kinesiologically based movement patterns. We derived a set of typical tasks for an experiment that emphasize various movements of the head, eyes, arms, wrist, torso and legs [22] (see Figure 1).
2. Focused tasks for data collection: The participants were engaged in the task of placing the red balls into the cylindrical containers (see Figure 1) and the green/blue cubes into the rectangular enclosure. These tasks can be broken down primitively into elliptical movements on the *XY*-axis and rotational movements on the *XZ*-axis. We tracked and recorded various data from the head-mounted-display (HMD) VR device, an eye gaze tracker (GAZE) and the hand-held-controller (HHC) as the users interacted and moved items within the VR environment (see Section 4.2.1 and Section 4.6).
3. VR identity model: The data derived from the task sessions were passed to a k-Nearest Neighbor (kNN) classifier to build an identity model, which was then used to identify the user during a future activity based on a preset threshold level of confidence (see Section 4.7).

## 2. Threat Model

**Figure 2:**
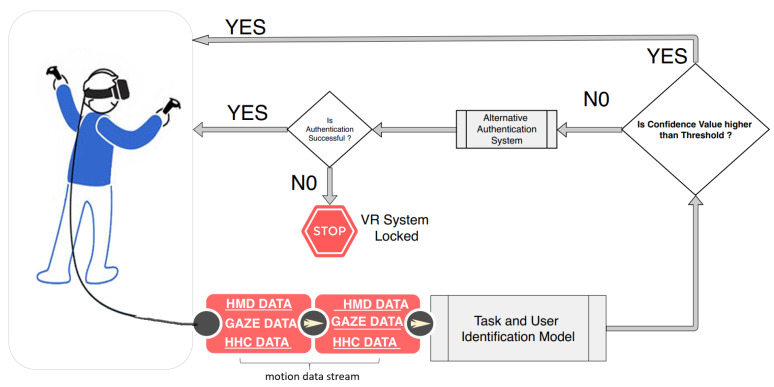
A diagram of the BioMove biometric identification process. As the user performs activities within the virtual reality environment, the motion data stream is passed to the Identification Model which determines the task and the user performing the task. A confidence value from the model is used to determine if the user is an authorized user. If the user is not an authorized user, the VR session, for example, can be stopped.

In an attempt to improve the usability of sensitive VR applications or processes within them, we identified three types of possible attackers. The *Type-I* attacker has no prior knowledge of the expected VR activities required and is mainly using a form of brute force attack. The *Type-II* attacker knows the required activity expected for identification (e.g., through watching a video clip or other observation methods), but has no other knowledge of the user’s features that serve to identify them. The *Type-III* attacker is the most dangerous attacker because the identification activity is known and this attacker has similar physical features as the valid user. Our real-time continuous identification system negates the possibility of malicious or opportunistic attacks when the authorized user is temporarily absent or distracted, thereby denying an attacker access to the VR environment. In our threat model, the valid user of the VR system steps away, and the attacker (*Type-II* or *Type-III*) who has previously observed or has prior knowledge of the VR systems starts to use the system. After a series of uncharacteristic head and body movements while interacting with the VR environment, our method will lock the device or prompt the attacker to prove their identity via an alternative identification method. Figure 2 gives a highly abstracted view of how such an identification system might be designed. A numeric value known as *confidence value* determines if the activity belongs to a valid user. The confidence *threshold* value and the number and nature of uncharacteristic movements triggering the secondary identification mechanism would in practice be tuned to strike a balance between usability and security. In Section 5, we performed a Whitebox penetration test using *Type-II* or *Type-III* attackers to determine the appropriate threshold confidence value. We make reference below to some examples of VR environments which might benefit from our methods.

*Virtual Reality Gaming Applications.* Numerous VR games are now becoming more multi-player oriented [8,23]. Many of these games require the user to perform different types of physically based interactions [24,25]; as such, an identification method such as BioMove would be very useful in these cases.

*Virtual Reality based Shopping Applications.* A growing number of online retailer and e-commerce platforms have started introducing virtual stores, allowing customers to transverse through virtual shops, pick and examine virtual 3D replicas of their product inventories [3,5,6]. These companies, such as Alibaba and Amazon, would benefit from a transparent identification system that continuously verifies in the background its current user based on the motion patterns they exhibit during their shopping sessions.

## 3. Related Works

Traditionally, identification has been a distinctively obvious and sometimes intrusive process, and the users are certainly aware of these processes. However, in recent years, as entertainment and payment activities are becoming more important and integrated into other activities, there has been a need to implement transparent non-intrusive methods of identification. This has led to a large body of research on non-intrusive methods but outside of VR systems, which this research attempts to fill. Transparent non-intrusive identification (TNI) is a process that attempts to extract identifying information from the user’s activities or from attributes freely exposed by the user while doing these activities, and therefore the system is able to continuously authenticate the user. Most TNI systems in the current literature use behavioral patterns such as gait [26,27], typing [28,29,30], eye movement [31,32], touch [33,34] and brainwave EEG patterns [35,36]. More recently, some research and commercial systems have harnessed users’ physical facial features and their finger biometric attributes to develop TNI systems. Although physical biometric attributes from users’ face and fingers are more accurate relative to behavioral biometrics, they are generally more intrusive to the users. As an example, for a single-point fingerprint sensor to continuously authenticate the user, the finger must pass frequently on the sensor, a requirement which might be considered intrusive or disruptive for certain tasks or users. Alternatively, user based behavioral attributes, such as touch patterns, gait and eye movement, that have no restrictive requirements and biometric data needed for identification can be captured transparently as the user types, walks or touches the system during an activity. We briefly review and describe important and relevant TNI studies below.

### 3.1. Eye Movement Biometric

Sluganovic et al. [37] developed an identification system that uses visual stimulus to elicit reflexive eye movements based on fixation, saccade and acceleration cues, in a predictable manner that supports the extraction of reliable biometric features. Using a gaze tracking device with a randomly generated stimulus to prevent replay attacks, the researchers achieved an equal error rate of 6.3%.

### 3.2. Head Movement Identification

Head movement and head pointing studies were performed by Li et al. [38], Saeed et al. [39], Li et al. [40] as a means to support biometric identification. Recently, an interesting research by Mustafa et al. [41] used only the sensors of an HMD to track users’ behavior patterns while they followed randomly appearing balls in a VR environment navigating only by head movements. They achieved an equal error rate of 7%. Furthermore, Li et al. [38] recorded a high degree of accuracy in identifying participants while they nodded when listening to music. Similarly, in a study by Yi et al. [42], a set of six head-explicit gestures that included making a circle, triangle, square and three kinds of lines were used to authenticate participants who used their nose as a pointer to perform gestures. The study obtained identification accuracy as high as 92%.

### 3.3. VR Movement or Task Driven Biometric Identification

A study by Kupin et al. [8] was similar to our work, but, in this study, their 14 subjects picked up and threw balls at a target within the VR environment using the HTC VIVE hand-held controller. They achieved an accuracy of 92.86% using a simple distance metric, which is much lower than the 98.6% accuracy we achieved with our method. Another interestingly similar study is by Pfeuffer et al. [23], which included HMD *integrated eye-tracking* and involved numerous activities such as *pointing, grabbing, walking and typing* within the VR environment in an attempt to capture biometric discriminants for identification. This study focused on the quality of the discrimnants and reported accuracies of at best 63%.

### 3.4. Contribution of Our Work

The above studies have some similarities to our work because they evaluated head or body movements within the VR space as a form of biometric discriminants. However, there are also significant differences that set our work apart.

- Biometric data sources: Mustafa et al. [41], Li et al. [38], Sluganovic et al. and Yi et al. [42] solely relied on head movements to gather the unique significant biometric features. This singular data input source increases the probability of malicious attacks. Our solution extracted biometric data from users’ head, eyes and hands in a kinesiologically conformative manner.
- Applicable to VR environments that are based on active body movements: Mustafa et al. [41] pointed out that their solution focuses on a particular VR environment, whereas our solution can be used to authenticate in any VR environment where kinesiologically active movements exist or are used. Additionally, we track head and eye movements, which are basic actions exhibited while using a VR system.
- Continuous vs. one-time identification: The solutions proposed by Li et al. [38] and Yi et al. [42] use a one-time identification model, allowing for a larger window of attack. Our study uses a continuous and dynamic identification model.
- Identification domain: The solutions of Li et al. [38] and Yi et al. [42] require the identification to occur in the physical domain via smart glasses and wearable devices, whereas our solution uses the virtual domain, such as any applicable VR experience for identification, making our application more scalable and adaptable.
- Multiple tasks: Kupin et al. [8] based their identification on a singular task of throwing the ball at a target, whereas our study involved six different VR tasks that include a variety of kinesiological movements.
- Penetration or vulnerability testing: Mustafa et al. [41], Kupin et al. [8] and Pfeuffer et al. [23] did not provide penetration testing of their solution. We conducted a *Type-II* and *Type-III* whitebox penetration testing (see Section 8) and our results indicated high resistance to these attacks.

## 4. Materials and Methods

### 4.1. Goals

In this study, our goal was to create a VR environment that allows users to perform a set of tasks. Our experiment comprised six different tasks, and each task was composed of a variable number of generic movements. We based these tasks on our review of the literature on kinesiology and VR applications, thus they represent elliptical curves in three-dimensional space involving coordinated motions of the head, eyes and hands. Using these tasks, we conducted a within-subjects study with 10 sessions per participant per day over a two-day period. A session took an average of 2 min. Our experiment provided the data that we needed to investigate and understand whether biometric discriminants can be extracted from motion data recorded from multiple sensors. Another expectation we had was for our resultant system to be able to easily identify its users and specific tasks.

### 4.2. Experimental Design

The participant movements were grouped into a set of tasks, which represent the *raw data* of our study and helped derive the *features* required in our analysis. The components of the movement and the resultant tasks are explained below.

#### 4.2.1. User Tasks

The movements were encapsulated into a task. We designed two variants of this task, one dealt with *balls* and the other with *cubes*. The participants were given the task of grabbing, transporting and dropping balls and cubes into containers strategically placed within the VR environment (see Figure 1). This ensures that kinesiologically valid motion data would be captured during our experiments. We then passed the collected data into a kNN classifier and trained an identification model with a high confidence value output. Below are the variables we tracked:

#### 4.2.2. The Raw Data Sources

- Task 1: Interacting with Balls in VR environment.
- Task 2: Interacting with Cubes in VR environment.
- Participant static metrics such as height, arm length and waist height.

#### 4.2.3. The Features Tracked

- Head-Mounted Display positional and rotational data.
- Eye tracking gaze positional data.
- Hand-Held Controller positional and rotational data.

#### 4.2.4. Movements

**Figure 3:**
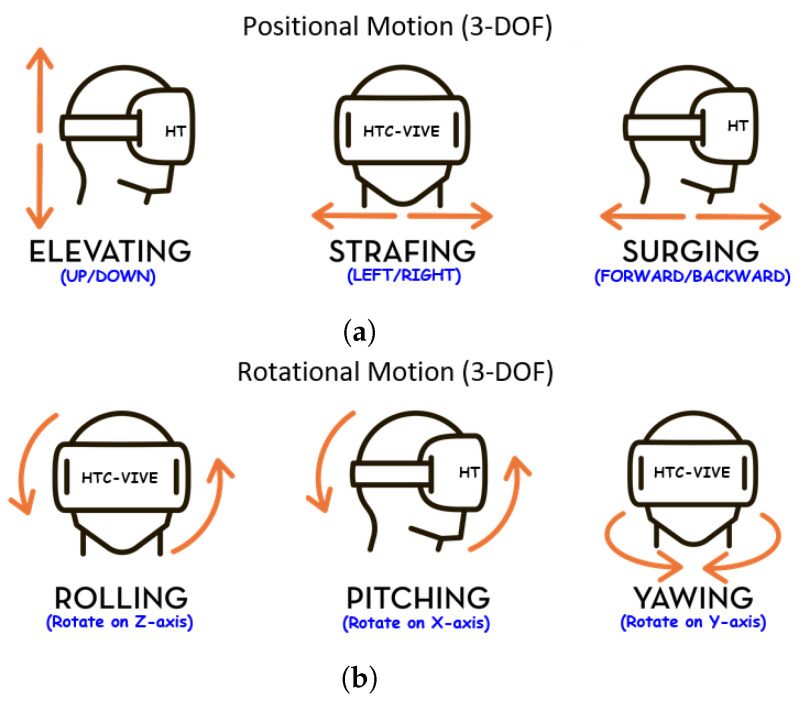
Possible 6-DOF VR Movements. (**a**) [Positional Motion] is the location of the object in the 3D world space. There are three possible positions motions (3-DOF). (i) Elevation is where the head/hand moves up or down *(i.e., when bending down or standing up)*. (ii) Strafe is where the head/hand moves left or right *(i.e., sidestepping)*. (iii) Surge is where the head/hand moves forwards or backwards *(i.e., when walking)*. (**b**) [Rotational Motion] is the orientation of the object in 3D world space. There are three possible orientations (3-DOF). (i) Roll is where the head/hand pivots side to side *(i.e., peeking around a corner)*. (ii) Pitch is where the head/hand tilts along a vertical axis *(i.e., when looking up or down)*. (iii) Yaw is where the head/hand swivels along a horizontal axis *(i.e., looking left or right)*.

**Figure 4:**
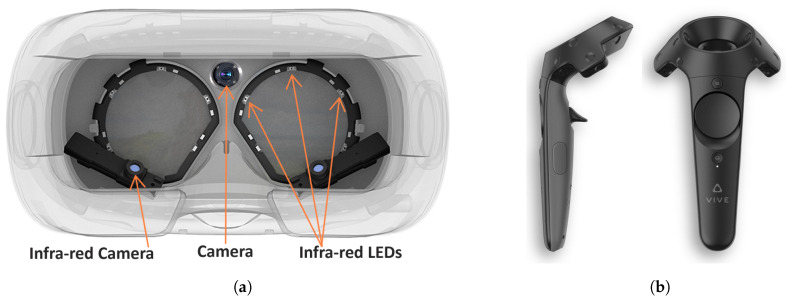
(**a**) The HMD upgraded for Gaze tracking: Interior view of a HMD device fitted with eye tracking hardware. (**b**) VR Hand-Held Controller (HHC) used in our study by participants to interact with VR objects with 6-DOF.

Each movement involves three main aspects of the body to varying degrees. These aspects are:

- Head Movements: The head moves through the possible six degree of freedom (6-DOF) while the participant targets, rotates and translates within the VR environment (see Figure 3). This was captured via the sensors in the HMD.
- Eye Movements: The eyes move as the participant locates, targets, grabs and moves the objects within the VR environment. This was captured using an eye tracking system embedded in the HMD (Figure 4a).
- Hand Movements: The hand moves through the possible 6-DOF while the participant grabs, transports, rotates, targets and drops the objects of interest (see Figure 3). This was captured via the hand-held controller (HHC) (see Figure 4b).

### 4.3. Research Ethics

All subjects gave their informed consent for inclusion before they participated in the study. The study was conducted in accordance with the Declaration of Helsinki. The protocol was reviewed by the XJTLU Research Ethics Committee and found to be low risk research. To promote reproducibility and transparency, all data and software from this study will be available on the GitHub repository at [43].

### 4.4. Apparatus

#### 4.4.1. The Virtual Reality Environment

**Figure 5:**
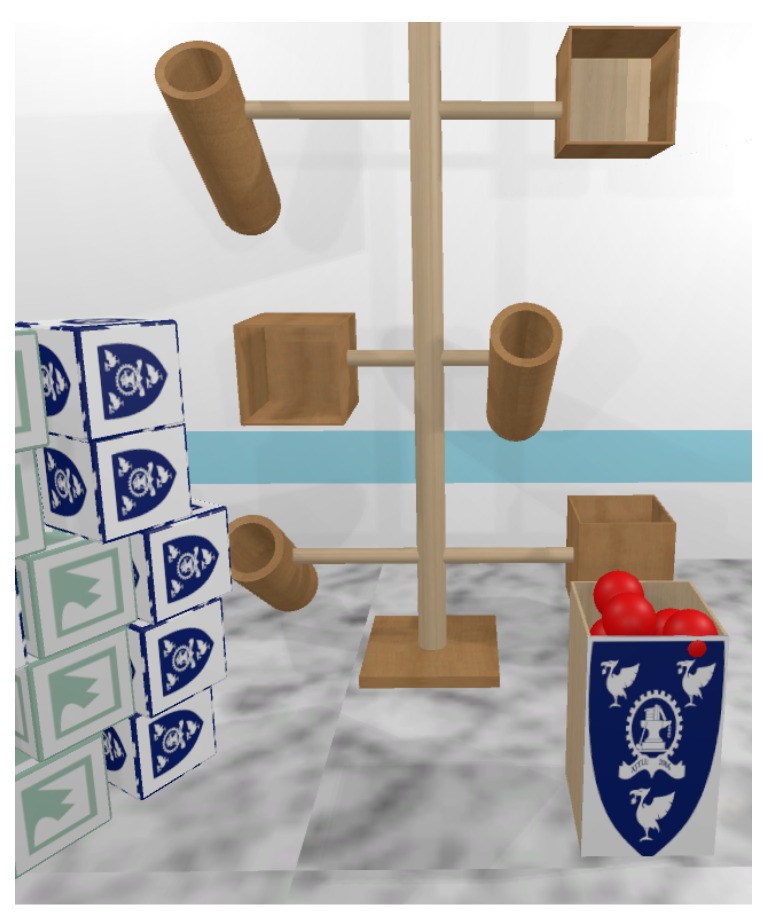
VR environment layout: The environment consist of a wooden stand, balls and cubes that participants relocate into the respective containers.

We created a VR environment, with Unity3D and C# on a Windows 10 PC *(Intel Core i7-6700, 128GB RAM, NVIDIA GeForce 1080)* to assist in the collection of our experimental data. In our VR environment, positioned in front of the participant is a wooden stand with three horizontal poles that are vertically spaced dynamically to be reachable based on the participant’s height (see Figure 5). Furthermore, at the opposite end of each horizontal pole is a cylindrical container and a cubic container that will be the final destination of the objects that the participants interact with during the session. Lastly, on the left side of the participant is a stack of cubes and to the right side of the user is a stack of balls. The VR environment mimics the real-world and the objects follow the laws of physics and their composite materials. The participant is allowed to move around within the environment.

#### 4.4.2. The Interaction Devices

The feeling of immersiveness of VR is due to the ability to interact realistically with objects within the environment. For this experiment we used the HTC Vive VR system. Our participants interacted using the following:

- Head: The VR headset containing the HMD has inertial motion sensors to estimate the participant’s head position and rotation. These sensors can capture data concerning the HMD and constantly update the VR environment according to head movements. The HMD allowed us to extract the positional coordinate and rotational, acceleration and velocity data for our experiment (see Figure 3).
- Eyes: The eye-tracking hardware, called aGlass [44], was installed inside the HMD to track the participants’ gaze as they performed the tasks. It can track the the positional coordinates (x,y) or direction where the participant is looking at within the virtual scene. The eye tracker was calibrated with a nine-point accuracy method (see Figure 4a).
- Hands: The hand-held controller (HHC) allowed participants to replicate their hand motion and usage within the VR scene to grab, move and release VR objects. The HHC also provided the positional coordinates, rotational, acceleration and velocity data during the experiment (see Figure 4b).

### 4.5. Participants

We enlisted 25 participants (11 female and 14 male) from a local university. Our pre-testing survey revealed that 72% of the participants were between the ages of 18 and 29 and only three participants were left-handed. All were aware of VR technologies, but only 64% of them had previously used a VR application. All participated voluntarily without any financial remuneration. Due to technical issues discussed in later sections, data from only 15 of the participants were used.

### 4.6. Task and Procedures

**Figure 6:**
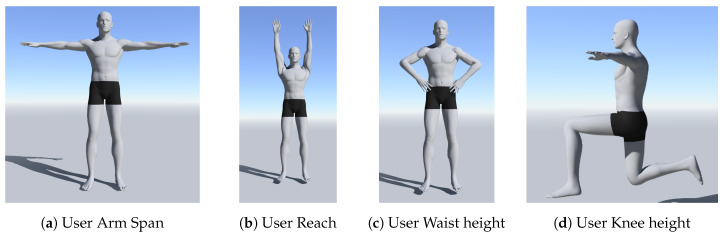
Pre-Experiment measurements: The Participants’ body metrics were taken before the initial experiment session commenced. The data were used to configure the VR environment to ensure that all participants would have similar experiences in the environment.

Each session started with a brief introduction to the experiment. Then, the participants were shown a 2-minute tutorial video that demonstrated the intended activity and how the VR devices were used, after which they were allowed to practice each task a couple of times. The physical measurement to capture the user height, arm span, waistline and knee height was done using the hand-held controllers as a measuring tool (see Figure 6). These measurements were used to configure the VR environment to ensure that all participants have similar experiences within the VR environment. For example, the VR objects are adjusted to the participants physical attributes so all items can be reachable. Additionally, eye tracking nine-point calibration was done once and the configuration data were stored with the participant ID for future use. A session took approximately 2 min, during which the participants would grab, relocate and drop three balls and three cubes into their respective containers (see Figure 1). Each participant was allowed to rest for 2 min after the completion of 5 out of 10 sessions.

### 4.7. Data and Feature Processing

We collected five features from the raw data consistently as the participants performed the tasks in the VR environment. We logged the data at the rate of *25 readings per second* (25 Hz), combining the data stream from the HHC, HMD and eye tracker to create a *movement data vector* with supporting metadata. This component movement data vector or ***movement vector*** is shown below:

- HHC and HMD:
  - Positional Data: x, y, z.
  - Rotational Data: x, y, z, w. *(Quaternion format)*
- Eye Tracking Device:
  - Positional Data: x, y.
- Miscellaneous Metadata:
  - Task performed: t, (where 1 ≤ t ≤ 6). *(We have six different tasks that are performed in a session.)*
  - Time Stamp: yyyy-mm-dd-hh-M-ss-zzz.

#### 4.7.1. Motion Data Resampling

In the experiment, each participant performed numerous sessions consisting of multiple tasks in the VR environment and their completion time for each task varied. Consequently, we needed to *resample* the data to create a consistent dataset for all participants. Each participant performed the task at different speeds while each task was defined by a sequence of movement vectors which were captured at *25 movement vectors* per second (25 Hz). For example, if participants A and B completed *Task 1* in 50 and 100 s, respectively, participant B would have 2500 movement vector readings, while participant A would have 1250 readings for the same identical task. Our resample process allowed us to select 1250 movement vector readings from participant B’s data, ensuring that all participants would have the same number of movement vector readings. Below are some definitions:

- Session: A Session **S** is a set of tasks **T** (see Equation (1)).
  (1) $$S=\{t_{1},...,t_{6}\}\;where\;cardinality\;\left|S\right|=6$$
- Task: A Task **T** is a set of movement vectors **m** (see Equation (2)).
  (2) $$T=\{m_{1},...,m_{n}\}\;where\;cardinality\;\left|T\right|=n$$
- Median: The median ***D*** of the cardinality of movement vectors |T| for each type of tasks $\left\{t_{1},...,t_{6}\right\}$ across all sessions **S** in the experiment are determined as follows: For Each Task type **$T_{x}$** (where 1 ≤ x ≤ 6) in the Experiment
  (3) $$D_{x}=Median(|T_{x}{|}_{1},...,|T_{x}{|}_{n})$$
  where $|T_{x}|$ is the cardinality of a set of movements of task type x.
- Resampling Process: The median or sample size $D_{x}$ was determined for each Task type $T_{x}$ (see Equation (3)). The Task movement count |T| was *resampled* to the relevant $D_{x}$ movement count. Therefore, at the end of the resampling process, each task group had movement vectors with the same cardinality, *which in this case is the median value* ***D**, across all participants.* Because resampled points will rarely, if ever, line up exactly with the original sample points, we used simple linear interpolation to calculate the value of the resampled point based on the values of the left and right nearest points in the original sequence. This allowed us to handle vectors that are both larger and smaller than the median size.

We collected data from 25 participants initially. We later had to remove the data of four participants due to invalid eye-tracking data, cause by a hardware issue. We also removed the data of six other participants due to inconsistent data from the HHC and HMD. This left us with 15 participants.

### 4.8. Machine Learning Classification Framework

We divided the data into 80% for *training* and the remaining 20% was reserved as *testing*. The classification was performed using the MatLab platform and, to gain insights into how different classifications methods might affect performance, we employed the built-in *MatLab Classification Learner App*, which automatically performed supervised machine learning tasks such as interactively exploring data, selecting features, training models and assessing results. Models tested included decision trees, discriminant analysis, support vector machines, logistic regression, kNN, naive Bayes, and ensemble classification. We also enabled the PCA (principal component analysis) feature to reduce the data dimensionality using PCA on the predictor data and then transformed the data before training the models. We selected the kNN classifier (k = 10) because kNN is a non-parametric, lazy learning algorithm and best suited for our movement vector datasets, in which the data points are separated into several classes to predict the classification of a new sample point. kNN is also very sensitive to outliers and bad features. We strongly believe the high-level of accuracy observed is due to our resampling process (see Section 4.7.1). Additionally, kNN is less computationally intensive and will work optimally on all types of low powered mobile devices.

Finally, after cross-validating each model type, the MatLab Data Browser displayed each model and its k-fold (k = 5), cross-validated classification accuracy and highlighted the kNN (k = 10) model as having the best accuracy of 98.6% among other classifiers. The goal of cross-validation is to test the model’s ability to predict new data that were not used in estimating it. With this goal in mind, one wants to estimate how accurately a predictive model will perform in practice, therefore a model is given a dataset of known data on which training is run (training dataset), and a dataset of unknown data against which the model is tested. The output is the predictive accuracy of the model.

#### Identification

For our identification scenario, we had to train on two different sets of predictors that resulted in models with different identification functions. We obtained two training models. The *first model* (the Task Identification model) focused on identifying the type of tasks $\left(t_{1},\ldots ,t_{6}\right)$ performed and the *second model* (the Participant Identification model) focused on determining the participants $\left(P_{1},\ldots ,P_{n}\right)$ performing the tasks. As the participants performed the task or motions within the VR environment, their physical actions or movements were broken or sampled into identifiable tasks by the *task identification model*. The output was then sent to the appropriate task focused *participant identification model*.

- Task Identification Model: This model is trained on identifying the task performed by the participants based of the movement vector streams. The resulting model makes a prediction of the task $t_{i}$ from the set of all possible tasks *T*. This stage allows a practical implementation where the results of this stage are used to select the correct participant identification model, thereby increasing accuracy and response time.
- Participant Identification Model: This model is trained to identify participants performing a particular task. The resulting model makes a prediction of the participant $p_{i}$ from the set of all participants *P*. In this scenario we have n=6 tasks, thus resulting in six different participant identification models. We argue that task specific model results in higher accuracy and faster identification systems.

## 5. Whitebox Penetration Testing

**Figure 7:**
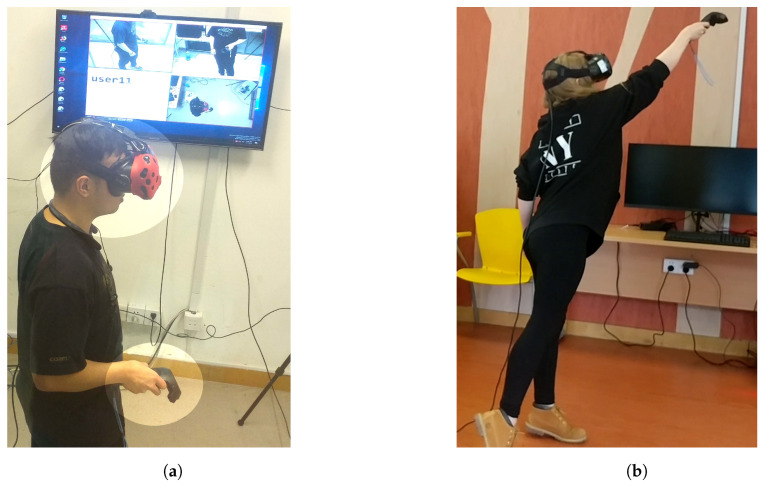
Task Sessions: (**a**) Participant performing a task while being recorded by three cameras placed at *TOP, LEFT and FRONT* locations. As shown in the picture the actions performed by the participant are monitored and recorded (see TV screen) with emphasis placed on the head and hand movements. This recording is later viewed by an attacker in an attempt to emulate the participant’s movement. (**b**) A participant stretches to maximum height as she performs the task. These kinesiological movements are captured and processed for unique biometric discriminants.

Penetration testing evaluates the security of a system against malicious attacks to identify vulnerabilities, and this requires us to use whitebox techniques. During the study, three high resolution video cameras were placed in strategic locations to record the external physical actions of our participants (see Figure 7). We selected one female participant, P6, and one male participant, P3.

**Figure 8:**
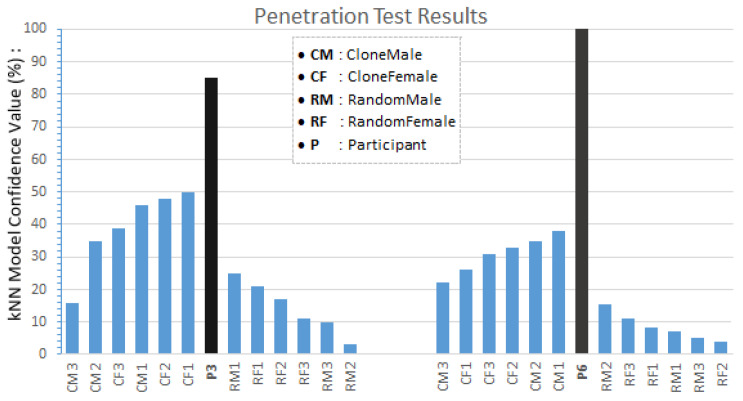
WhiteBox Penetration Test: Attackers mimicking the tasks performed by the valid Participants P3 and P6 in an attempt to breach the security of the BioMove identification system. Results indicate different level of confidence values shown above. Attackers with similar physical features to the valid users have higher confidence values.

Two groups of six attackers *(three females and three males)* were selected. The first group consisted of users who were similar in *height*, *arm span* and *weight (CloneMale* ***CM*** *or CloneFemale**CF**)* to the valid participant. The second group consisted of randomly selected users *(RandomMale* ***RM**or RandomFemale* ***RF**)*. Each attacker performed five sessions each of *Tasks 1–6* after watching videos of the two valid participants (P3 and P6) performing these tasks. We passed the captured attacker movement task data into the kNN model trained for P3 and P6 and tracked the results based on the level of confidence values (see Figure 8). As shown in Figure 8, the attackers did not attain the minimum confidence value required to qualify as a valid user. We also observed that the attackers with similar physical features as the valid users had higher confidence levels; these levels are even higher when attackers and valid users are of a similar gender.

## 6. Results and Discussion

### 6.1. Identification Performance Evaluation

**Figure 9:**
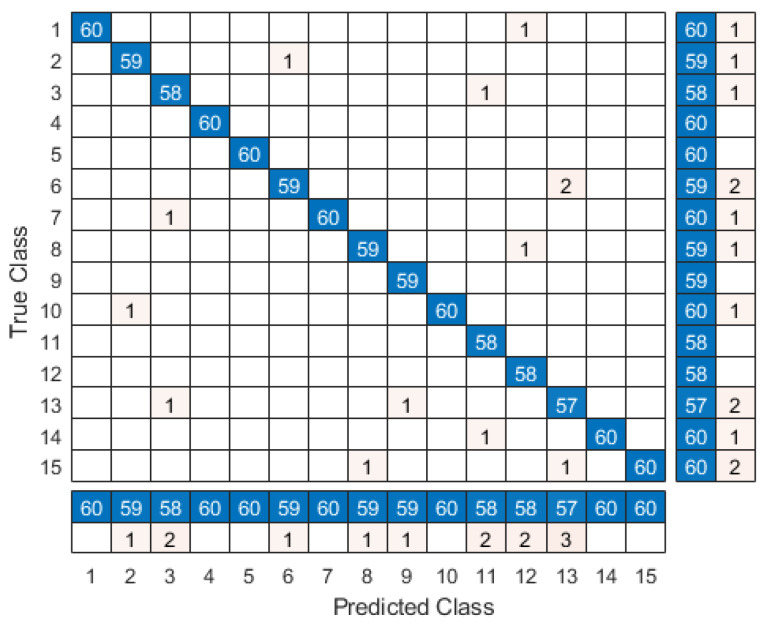
kNN Classifier Confusion Matrix: A confusion matrix summarizes the performance of a classification algorithm. It gives a better idea of what your classification model is getting right and what types of errors it is making. Classification accuracy alone can be misleading because we have more than two classes in our dataset.

**Figure 10:**
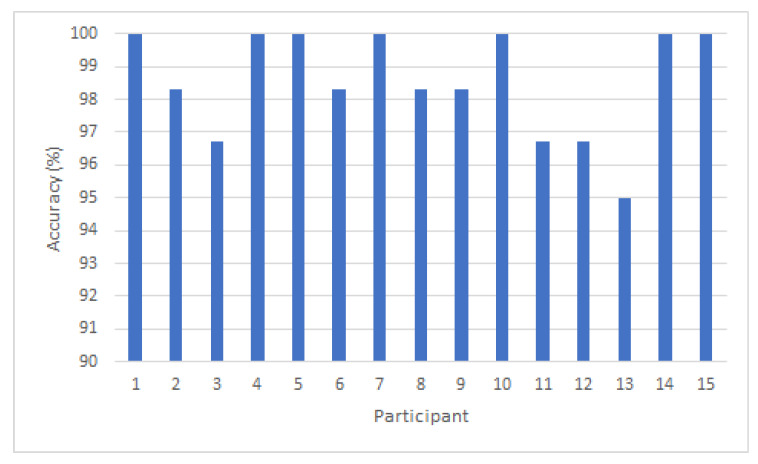
Accuracy per participant across all tasks: The prediction accuracy of participants movements across all tasks.

We achieved highly accurate results in identifying participants within the VR environment. The identification process involves first identifying the task and then identifying the participant. On the Windows 10 PC (Intel Core i7-6700, 128GB RAM, NVIDIA GeForce 1080), we clocked the GPU down to 1600 MHz and observed an average identification processing speed of 0.035 ms per classification. At this speed, the response is instantaneous from the user perspective. Overall, we achieved a cross-validated classification accuracy of 98.6% and an error rate of 1.4%. We generated a confusion matrix that summarizes the actual performance of the kNN algorithm, thus providing us with a view into what the model handled correctly and what types of errors it was making (see Figure 9). Furthermore, results from the movement data identification of all participants across all tasks is shown in Figure 10. Our whitebox penetration test indicates that attackers impersonating the valid participants resulted in less than 50% accuracy value, thus an accuracy value threshold above 80% will be sufficient to protect malicious attacks producing false positive identifications. The penetration test also confirms that attackers who are physically similar to the valid participant produce higher accuracy values (see Figure 8).

The potential application of this research is a form of universal identification module that is hosted by the VR device firmware system and independent of any specific VR software application, be it a game or productivity tool. This solution may be implemented using the low-level Open Broadcaster Software (OBS) OpenVR [45] application programming interface (API) kit or the new OSVR [46] input plugin that mirrors all movement data at the hardware level and dynamically hooks into base GUI modules of all high-level VR applications to interrupt their session if the transparent non-intrusive identification (TNI) based on these movement data fails (see Figure 2). This proposed universal identification module can also be configured to trigger the local identification process on the high-level VR session it interrupted as a means of providing a second-factor identification and allowing the system to request the user to provide an alternative identification. Our research was aimed at users between the ages of 18 and 30, which is the largest demography of user groups of VR systems [47]. Although looking at the aging process and how that affects the use of the VR identification system is interesting and important to explore, so that we can have more targeted systems for each specific group, it is outside of the scope of the paper, therefore a part of our future work.

### 6.2. BioMove as an Authentication Mechanism

While our work was mainly focused on using BioMove as a non-intrusive, continuous identification mechanism, another use for it would be as an authentication mechanism. Authentication differs from identification in that, while identification classifies the candidate as being a member of fixed set classes, authentication can also reject a candidate as not being a member of any class. To extend our method to authentication, we need to develop this criteria for rejection.

Our classifier is kNN, which selects the class of the input based on its nearness to other members of the class, thus we chose our threshold in a similar manner:

$$\forall C_{j}:thresh_{j}=\max_{x_{l},x_{k}\in C_{j}}∥x_{l}-x_{k}∥$$

where $C_{j}$ are the classes corresponding to the valid users and $x_{k}$ are the vectors belonging to $C_{j}$, used to train the kNN model. Our rule for rejection for the presented example, $x_{new}$, which is provisionally assigned to class $C_{select}$, is to reject if and only if:

$$\min_{x_{k}\in C_{select}}∥x_{new}-x_{k}∥>thresh_{select}$$

Intuitively, what this means is simple. A kNN model is just a collection of vectors and their class labels. Classification is done by computing the distance between the new vector and the existing vectors in the model, and allowing the nearest k neighbors to vote for the class label. Our rejection method simply says that, if the distance between the new vector and the nearest neighbor of the tentatively assigned class is larger than the maximum distance between any two class examples in the model, then reject.

To evaluate this system, we trained the model to recognize a single user, using five data points to train and the remaining five data points for that user, along with the data points from the other users, to test. We repeated this 105 = 252 times for each user (once for each possible training set for that user) and calculated the average over all users for the false positive (classifying the presented data as belonging to a valid user when the do not) and false negative rates (rejecting a valid user).

For our data, we found the false positive rate to be 0.0032% and the false negative rate 1.3%. This suggests that our threshold selection is strongly biased towards false negatives. Again, intuitively, this makes sense, since we are conservatively rejecting even valid samples if they fall outside of the variability of the training data. For authentication tasks, this is appropriate, since allowing an invalid user access to the system is significantly worse that rejecting a valid user. While we are certain this threshold computation could be improved, this is outside the scope of this work.

## 7. Limitations and Future Work

Because our research was primarily focus on one of the most common user groups (i.e., those between the ages of 18 and 30), we only recruited participants in this category. In the future, we plan to extend our research to different groups and evaluate the relative accuracy and robustness of our approach across multiple groups, especially taking into account the effect of aging on the kinesiological movements of users. In addition, future work will explore integrating our solution into pre-existing VR environment frameworks when VR hardware manufacturers provide APIs that allow us to embed our systems into their firmware. As a result, we will have a real-time application that will be totally responsive and transparent to users while interacting with a VR device doing typical tasks such playing a game, doing learning activities, and exploring multi-user worlds. We believe that our research can serve as a second-factor authentication system where the verification will be 1-to-1.

## 8. Conclusions

Our study provides a preview into what can be achievable in terms of using kinesiological movements within virtual Reality (VR) environments for biometric identification. We evaluated 65,241 dataset of head, eyes and hand movements using machine learning to create a continuous biometric identification system. In our best configuration, we attained a classification accuracy of 98.6%. These results indicate that the head and body movement based biometric identification holds promise that points to the possibility for continuously identifying and authenticating participants in VR systems. In practice, our method would not have to be VR application specific since we have distilled the primitive body movement patterns that are similar across different VR environments. For example, a movement biometric template captured in the core VR systems can be used to authenticate in a VR gaming application or banking application. We also determined that attacks on our BioMove identification system would require a highly sophisticated attacker. We argue that simultaneously providing the system false-positive data vectors such as eye, head and hand movement vectors that are kinesiologically replicating a valid participant is extremely difficult. 

## Abbreviations

The following abbreviations are used in this manuscript:

TNI	Transparent Non-intrusive Identification
VR	Virtual Reality
EEG	Electroencephalogram
KNN	k-Nearest Neighbor
HHC	Hand Held Controller
HMD	Head Mounted Display
HCI	Human Computer interaction
DOF	Degree Of Freedom
FAR	False Acceptance Rate
FRR	False Rejection Rate
EER	Equal Error Rate
OBS	Open Broadcaster Software
OSVR	Open Source Virtual Reality
API	Application Programming Interface
